# Partial Extraction Therapy for Implant Placement: A Newer Approach in Implantology Practice

**DOI:** 10.7759/cureus.31414

**Published:** 2022-11-12

**Authors:** Mohd Sohail Ahamed, Bhushan P Mundada, Priyanka Paul, Amit Reche

**Affiliations:** 1 Oral and Maxillofacial Surgery, Sharad Pawar Dental College and Hospital, Datta Meghe Institute of Medical Sciences, Wardha, IND; 2 Public Health Dentistry, Sharad Pawar Dental College and Hospital, Datta Meghe Institute of Medical Sciences, Wardha, IND

**Keywords:** pet, pontic shield, implant, root submergence, socket shield, partial extraction

## Abstract

One of the most popular treatment modalities in routine implantology practice is extraction followed by immediate or delayed implant insertion. Teeth removal alone is insufficient, particularly in the maxillary anterior region of the jaw. Patients may experience several issues after tooth extractions. Due to trauma and the loss of periodontal ligaments, post-extraction alveolar ridge resorption cannot be prevented. Atraumatic extraction, socket preservation, grafting, and implant placement immediately after the extraction are some of the procedures that are carried out to minimize or prevent the resorption of alveolar bone. Osseointegration is essential for keeping the clinical effectiveness of dental implants. If the supporting tissues at an implant site resorb and are worsened by risk factors for recession, there may be considerable esthetic and functional failure. Implant placement at the retained root structure preserves the buccal bone resulting in an excellent emergence profile. Resorption in the posterior alveolar ridge may result in a decrease in attached keratinized tissue and a decrease in vestibular depth. This might have a negative impact on the stability of the implant and leads to peri-implantitis resulting in the failure of the implant. Without papilla loss or arch collapse, partial extraction therapy has resulted in effective esthetic outcomes. The socket shield technique is a minimally invasive surgical procedure that helps to maintain both soft and hard tissues by preserving a small section of the root. It lessens the necessity for surgeries on bone and mucogingival grafts, cutting the length of the overall recovery process and reducing the treatment time. When soft and hard tissue grafts are used to fill the socket before applying pressure with pontics, it is known as the pontic shield procedure. However, there is no published study that explains partial extraction therapy in a straightforward and clear manner that can guide a practitioner in determining a shield design with a proven track record of success. This review article focuses on the partial extraction procedure which is very helpful for preserving soft and hard tissues in cases involving immediate implant insertion post-extraction. It has long-term therapeutic success with implant and pontic therapy. This review article will also be helpful for clinicians to understand shield design in different case scenarios and help to learn step-wise procedures carried out in partial extraction therapy.

## Introduction and background

Tooth extraction or removal has continued to be a major and popular treatment option in general day-to-day dental practice. Nowadays, there is a tremendous demand for esthetics due to various factors such as development in lifestyle, increase in literacy rate, and increase in desire. Everyone wants to appear attractive and have an appealing and beautiful smile. Therefore, only tooth removal (extraction) is not enough, especially in the area of esthetics (anterior maxilla). The most crucial and pressing issue is replacement and rehabilitation. To replace lost teeth or tooth with a poor prognosis, dental implants have become the standard. Dental implants were initially primarily utilized to attach complex poly-unit prostheses, but in recent years, single-tooth replacement, particularly in the esthetic region, has become quite successful. For an implant's long life and clinical success, osseointegration is the foremost requirement. The osseointegration term coined by Dr. Branemark is a clinical procedure that required time for healing and binds firmly to the bone graft material resulting in asymptomatic during functional stresses and is thought to be a prerequisite for implant placement [1,2]. However, in recent years, patient satisfaction has come to depend more on the esthetics of the restoration which is due to the osseointegration of a dental implant [3,4]. To generate an implant-supported restoration that looks natural, some authors have emphasized the importance of presurgical planning [5], while others have put more of an emphasis on proper implant placement [6,7]. To produce a smile that blends the patient's face and expectations, one (clinician) should take into consideration the principles of gingival and dental esthetics [3].

The need for maintaining both hard and soft tissues has grown along with the constant pressure to produce cosmetically attractive results. Resorption of alveolar bone after dental extraction and implant placement can be a serious problem with often extremely poor esthetic effects. Numerous investigations have shown that bone resorption has been documented up to an average width of 50%. Height loss of 2-4 mm, or an average of 1.24 mm, has also been observed [8]. This resorption procedure, however, is quite unpredictable [9]. Alveolar ridge volume is significantly reduced by 0.5-1% as a result [10]. After tooth extraction, the thickness of the alveolar bone decreased by 3-3.5 mm, especially on the buccal aspect of the maxillary front region [11]. Within one year of tooth extraction the alveolar width, on an average of 5-7 mm, is reabsorbed. As the buccal plate is thinner and weaker in structure, two-thirds of its resorption occurs in the first three months [12,13], as a result of which, after tooth extraction, there is a difference in the height of the buccal and the lingual plate of the resorbed ridge [14,15]. The loss of the periodontal ligament (PDL) due to trauma during extraction is the major contributor to the change, and several strategies and therapies are developed to halt the resorption of alveolar bone. Immediate implant placement, atraumatic extraction, socket preservation procedures, and placement of various grafts maintain the dimensions of the alveolar ridge by preventing the collapse of cortical plates. These methods are frequently used to reduce bundle bone loss. Although studies have not shown that these procedures completely preserve the alveolar socket, they do have a considerable impact on keeping the post-extraction alveolar bone [16].

Understanding the periodontium and the post-extraction loss of tissues is related to the fundamental procedure of tooth removal, which involves cutting off the highly vascular PDL system that supplies blood and nutrition to the bundle bone [17]. Resorption of the socket after extraction is therefore inevitable. If the supporting tissues at an implant site resorb and are worsened by risk factors for recession, there may be considered an esthetic and functional failure [18]. According to research, if the tooth root is still inside the alveolar process, there is very little bundle bone resorption. Keeping this in mind, partial extraction treatment (PET), a sequence of precollapse procedures that utilize the tooth itself to compensate for the loss of alveolar tissue, came into existence, and addressed issues caused by extraction. The PDL-bone complex as well as its vasculature is kept intact by preserving the tooth root attachments to the bone [19]. To understand the viability of the implant put right after the PET and to make a prediction regarding its clinical success, this article will be helpful. The main goals are to describe PET and explore it and to simply and carefully review it, and explore procedures carried out in its various types, whether this technique provides long-term clinical success in implant and pontic treatment, and whether PET enhances the functional and cosmetic requirements.

## Review

Dental implants have become an inseparable part of modern dentistry. Dentures and bridges were used before dental implants, but because of the procedure's proven track record of success, reliability, and relatively fewer complications, dental implants have become a highly popular alternative treatment option. By the end of 2023, the market for dental implants is projected to have grown to around 1,070,996,912.54 Indian rupees. Although it has been shown that dental implants have a survival rate of more than 90% during a 10-year monitoring period, this presents a challenge for the future. The population's aging is a factor in this issue. The number of old individuals is significantly rising globally. Although tooth loss is increasingly common as people age, elderly people are also more vulnerable to environmental influences that might harm their physical health. Conditions like diabetes, osteoporosis, obesity, medication usage, etc. can lead to failure of bone regeneration around the dental implants (osseointegration). This ultimately results in implant failure and due to which the success rate may be impacted. Considering the aforementioned, it is obvious that dental implants need to continue to advance to prevent failure and to have long-term success in replacing the natural tooth. Various techniques like forcefully directing abrasive particles at a high speed, coating the implant with a plasma spray, application of acid on the implant surface, electrolytic passivation, and biomimetic coating have been used over the implant surface to make surfaces rougher and this facilitates osseointegration. Apart from this, very few modifications have been done in relation to an intentionally removed tooth that might prevent bone loss and that can act as a nidus for osteointegration of the implant.

Partial extraction therapy

After a tooth is removed, the blood supply to the periodontium's thin bone walls is cut off, which causes the labial cortical plate to resorb [13]. It suggests that preserving a root may influence how often frontal bone resorption occurs. Numerous studies demonstrated that the alveolar bone may be preserved by keeping the decoronated root, which is crucial, or endodontically treated using the root immerse approach [20]. It has been suggested that maintaining the tooth root or a piece of it will keep all the tissue components of the periodontium together by preserving the PDL fibers and the vascular system that nourishes the bundle bone and holds it. Many researches have been conducted to demonstrate various ways for preserving residual ridge when resorption begins following tooth extraction. The latest study has also shown that implant placement at the preserved root surface preserves the buccal bone, resulting in an excellent emergence profile [21].

**Figure 1:**
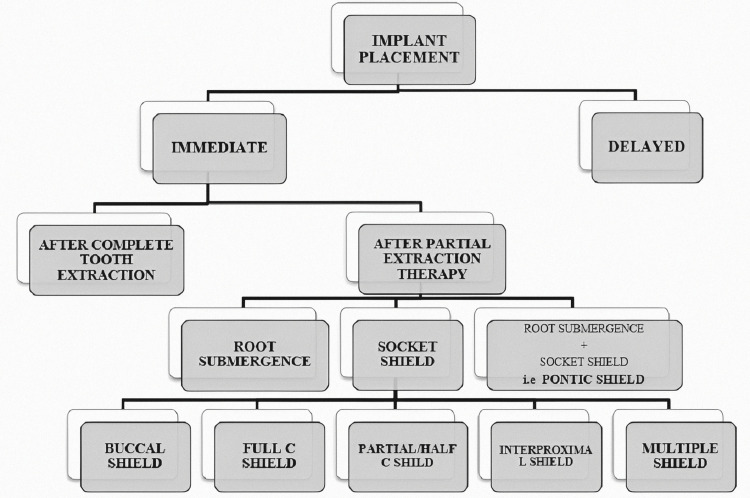
Classification of Implant Placement Options Based on Clinical Conditions, Radiographic Evaluation, and Patient Requirements

Based on the duration of treatment, implant placement can be divided into two types (Figure 1): immediate or delayed placement of the implant, which occurs after the extraction socket, has healed completely. Immediate implants have recently become popular, which implies that implants can be inserted right away following a full tooth extraction or following PET. 

**Figure 2:**
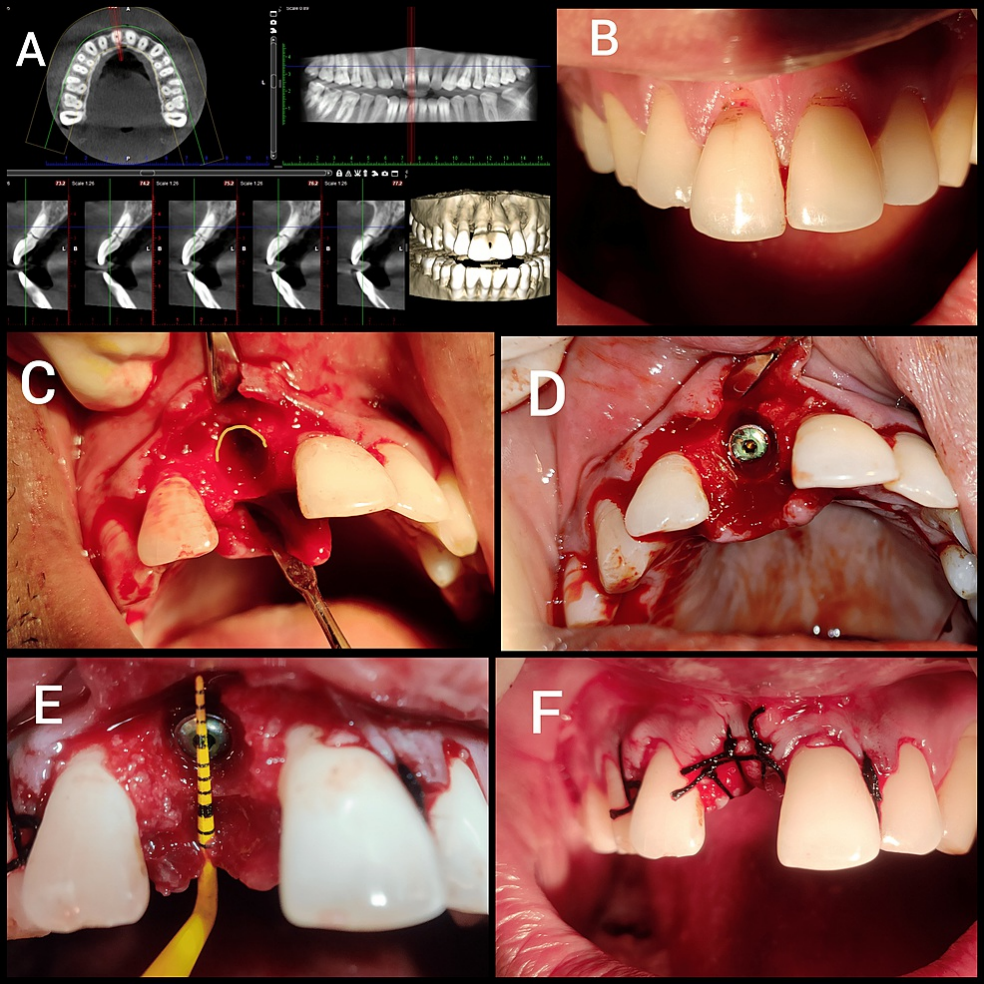
A) Horizontally Fractured Central Incisor on CBCT. B) Clinical Presentation of Right Central Incisor. C) Extraction Socket Showing the Position of Shield. D) Placement of Immediate Implant. E) Socket After Implant Placement. F) Closure of Surgical Site for Healing. CBCT, cone beam computed tomography.

Traditionally, intraoral periapical (IOPA)/orthopantomogram (OPG) (two-dimensional [2D] imaging) is used for treatment planning. Cone beam computed tomography (CBCT) is recently in trend because of its high efficiency and reliability for identifying bone density overall treatment plan before and after the implant placement either immediately or delayed. After tooth extraction, the direct implant is placed inside the socket in the proper position with a bone or soft tissue graft material if required and sutured back for healing. With time researchers have proven immediate implant to be efficient even after bearing masticatory load. But studies have also proven PET to be more effective than other current practice techniques for implant placement. Histological examination reveals formation of new cementum, when clinician intentionally left a portion of tooth or tooth root inside the alveolus acting as a shield to protect the implant (Figure 2). Various modifications have come up based on clinical scenarios and developing knowledge about the materials and upcoming studies based on long-term clinical success.

Root submergence

**Figure 3:**
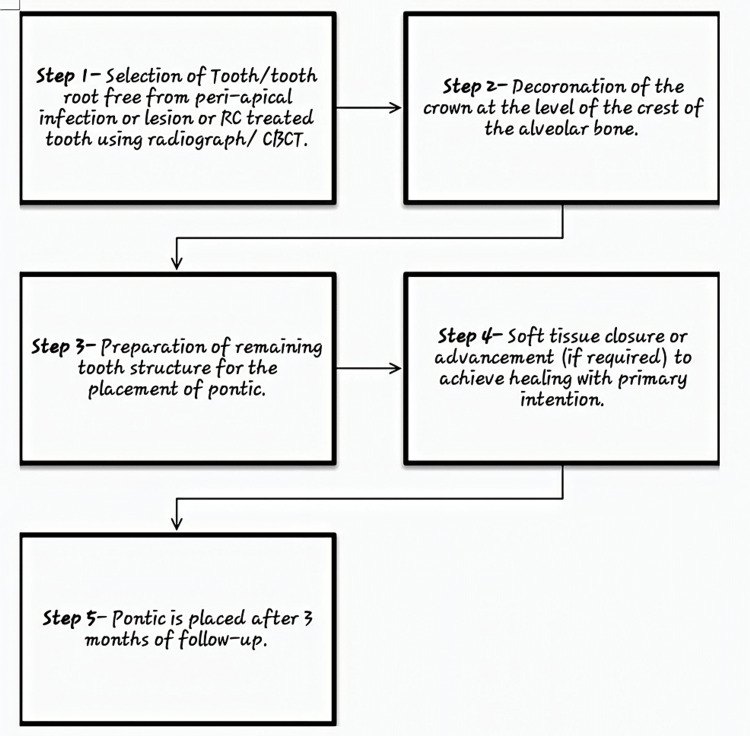
Represents Procedural Steps in Root Submergence Technique CBCT, cone beam computed tomography.

**Figure 4:**
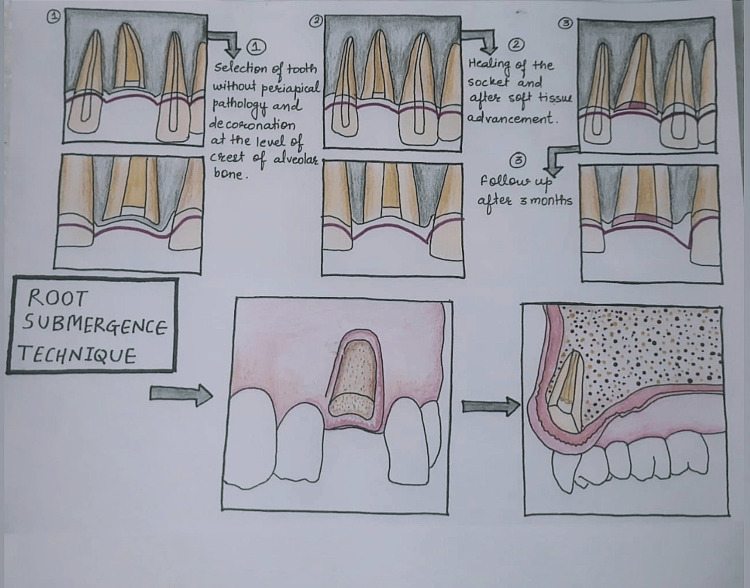
Diagrammatic Presentation of Root Submergence Technique

Root submergence was first created to limit the deterioration of the remaining alveolar ridge and to enhance the preservation and robustness of the denture [22]. Clinical as well as histological studies have shown that the residual ridge may be successfully preserved by endodontically treated or non-infected teeth roots that are free from peri-apical pathologies and that are decoronated at the level or below the level of crest of alveolar bone and totally buried inside the alveolar bone [23-25]. This idea has recently been utilized in the rehabilitation of patients wearing fixed dental prostheses [20,26-28]. The procedural step in the root submergence technique (Figure 3) differs from other methods performed in PET by complete preservation of the root structure without sectioning it or without any modification (Figure 4). Also, the preserved root structure should be either free from periapical infection or root canal treated. But the core concept of preservation of periodontal fibers and their blood supply to prevent the loss of other components of periodontium remains the same. Additionally, implant-supported prostheses have also used the root submergence method in the upper front region of jaws to achieve an esthetic and pleasing profile [20,27,28]. Without arch collapse and loss of papilla, the root submergence treatment has yielded successful esthetic results, but long-term results have not been studied. 

Socket-shield technique

**Figure 5:**
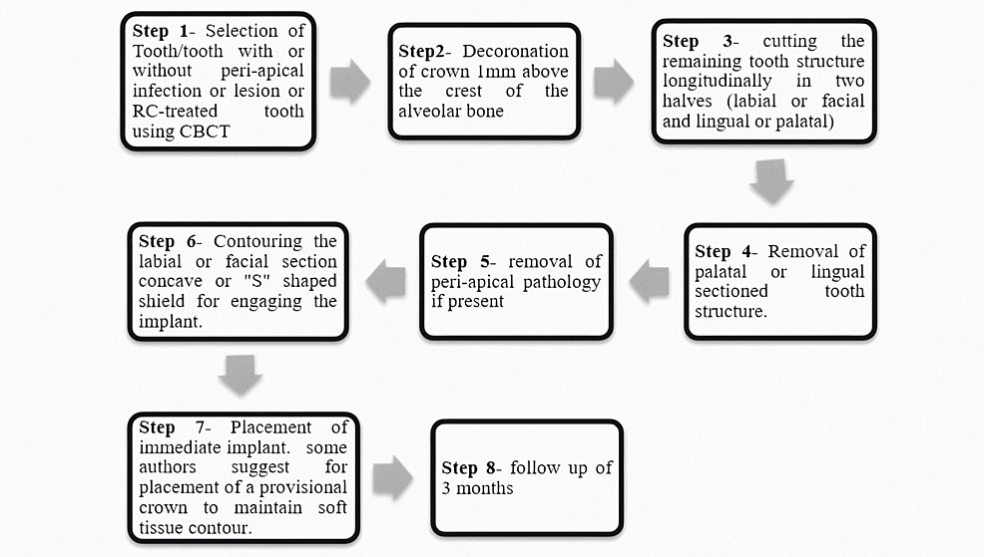
Represents Procedural Steps in Socket-Shield Technique

**Figure 6:**
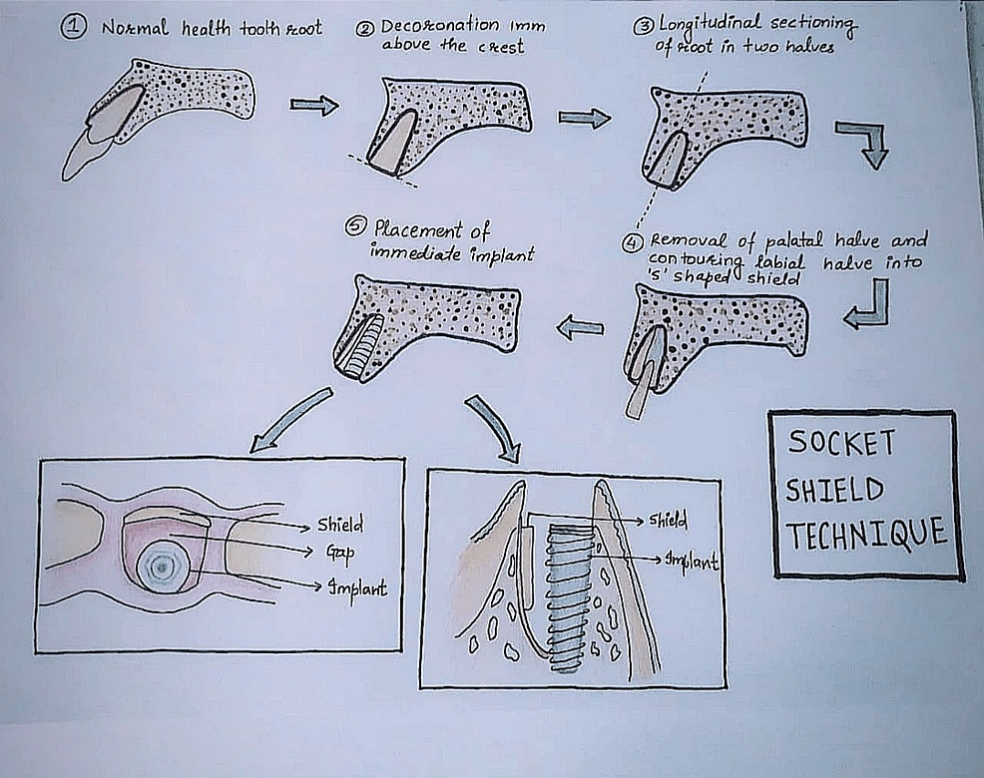
Diagrammatic Presentation of Socket-Shield Technique

A study on a beagle dog, proposed by Hürzeler et al. (2010), showed the socket-shield technique suggesting that partial/incomplete retention of tooth roots is done to avert the buccal bone from resorption. He conducted a mandibular premolar hemisection while keeping a buccal portion of the distal root that was above the crest of the alveolar bone by 1 mm. Without making contact with the intentionally left root surface, the implant was positioned lingually to the retained root fragment. There were no problems, and the histologic analysis revealed that in between the implant (titanium) and the retained root segment there is a development of new cementum, which is an excellent indicator for retention and stabilization of the implant [21]. A piece of the root is preserved through a minimally invasive surgical treatment called the socket-shield method, which helps in maintaining natural soft and hard tissue forms. The overall duration of the therapy is shortened since fewer procedures like soft and hard tissue grafts are avoided. The interdental papilla can be conserved even in situations with proximity implants by creating an interdental socket shield, as seen by Kan and Rungcharassaeng [29] and after them Cherel and Etienne [30]. Procedural steps in the socket-shield technique as in Figure 5 show a similar procedure that is carried out in clinics where the root portion is preserved 1-1.5 mm above the alveolar crest and then sectioned, and then the preserved buccal root structure is made into an "S" shaped structure after removal of peri-apical pathology if present; Figure 6 diagrammatically represents it. Socket-shield technique has a very promising procedure for preserving pink and white texture, preventing the exposure of implants, and offers treatment for cases where cosmetic elegance is crucial, including high lip lines and maxillary anterior. This treatment not only safeguards but also helps to maintain bone and gingival tissue form in the future, so long as the shield is intact.

Socket-shield technique is further subdivided into various types depending on the clinical condition as shown in Table 1.

**Table 1:** Classification of Socket-Shied Technique Based on Clinical Condition

Classification	Definition	Indication
Buccal shield	Shield (i.e., preserved root structure) is present only on the buccal surface of the extraction socket.	An edentulous area bounded by the tooth structure on both sides.
Full "C" shield	Shield (i.e., preserved root structure) is present on the buccal surface as well as on the mesial and distal surface of the extraction socket.	An edentulous area that is bounded by the implant on both sides.
Partial C shield	Shield (i.e., preserved root structure) is present on the buccal surface as well as on either side, i.e., either distally or mesially of the extraction socket.	An edentulous area that is bounded by the implant on one side.
Interproximal shield	Shield (i.e., preserved root structure) is present on both sides (interproximally), i.e. distally and mesially of the extraction socket.	An edentulous area that is bounded by the implant on both the sides and the buccal side requires a graft.
Multiple shields	Shield (i.e., preserved root structure) is present only on the buccal surface of multi-rooted teeth.	An edentulous area of multirooted teeth, i.e., molars and premolars.
Note: In the above classification, shield should always be present on side at which the implant is placed previously, i.e. adjacent to it.		

Pontic-shield technique

In a procedure akin to the socket-shield technique, Gluckman et al. [31] proposed the pontic-shield technique, in which they filled the socket with bone graft material taken from different species before the application of pressure by pontics. Thirteen of the cases in the series that were shown were successful, while one case had to have the advancement flap reshaped because the shield had been exposed. They draw a conclusion from their study by characterizing the method as a workable choice for alveolar ridge safeguarding in pontics and assuming the necessity for further research in this area. The pontic shield is advised in cases with peri-apical pathologies or the failure of endodontic (root canal) therapy where root submergence is contraindicated and for areas intended to receive restorations, such as a fixed partial denture supported by teeth or implants or removable partial denture [31]. The pontic shield integrates the socket-shield method with socket grafting procedures [32]. First, the socket shield is ready. It is taken into consideration that all the stages outlined above, including treatment planning, CBCT, and radiographic imaging, are repeated exactly. The entire apical infection must be removed from both the root and the root's apex. Magnification and illumination are required for this process.

The procedural steps for the socket-shield technique are almost similar to the pontic shied technique with the introduction of an osteoconductive material, i.e. material derived from other species in the socket and on top of it soft tissue graft of the same patient before pushing the ovate pontic against it.

After Hürzeler et al., in the studies conducted in the years 2016 [19] and 2017 [33], the authors described PET in a more comprehensive and precise manner where they categorized the PET into three categories: All of the following procedures - root submergence, socket shield, and pontic shield - require the absence of apical pathology to be successful: i) root submergence, which involves keeping the complete root under the gingiva to preserve the actual volume of the root; ii) socket shield, which involves simultaneously placing the implant on the palatal surface of the socket and preparing the buccal surface of the root and by 1 mm above the bony crest; and iii) filling the gap between the implant and preserved tooth structure by using an osteoconductive material before pressing an ovoid pontic, and a three-month follow-up and soft-tissue sealing were further advised. The study's conclusion emphasizes that partial extraction techniques as a conservative approach should be considered by practitioners for preserving bundle bone and maintaining residual ridge height in oral rehabilitation [19].

PET is not only used for the anterior region but also for the posterior. The posterior ridge may lose its functional and esthetic significance if the alveolar ridge is considerably decreased. More importantly, posterior bone loss may result in the lack of attached keratinized tissue and a decrease in vestibular depth [34]. Such alterations may have a negative effect on the implant's functional stability and lead to peri-implantitis when coupled with insufficient alveolar bone [35]. It may be necessary to have surgery for the correction of the vestibule, guided tissue regeneration, mucogingival surgeries which include grafts, and/or sinus augmentation in the case of the maxillary posterior region. These procedures call for a high level of competence and are not without complications [36,37]. Documentations and case reports have shown good results in the molars that are treated using PET as well.

## Conclusions

Clinical professionals all over the globe are becoming more and more interested in PET. In cases involving immediate implant insertion post-extraction, this therapy has shown very promising results in preventing alveolar ridge resorption. This review article will give readers the knowledge they need to comprehend partial extraction in detail, including its various types, their indications, the steps involved in performing PET, and how to design a shield (preserved root structure) that will deliver the best esthetics, even in cases involving immediate implants, as well as meet both functional and cosmetic needs of the patients not just in the anterior but also in the posterior region of the jaw with demonstrable results. After rehabilitation with PET, there are retrospective pilot studies and extensive literature-based data that show long-term therapeutic effectiveness with implant and pontic treatment.
